# Irreversible Electroporation (IRE) in Locally Advanced Pancreatic Cancer: A Review of Current Clinical Outcomes, Mechanism of Action and Opportunities for Synergistic Therapy

**DOI:** 10.3390/jcm10081609

**Published:** 2021-04-10

**Authors:** Zainab L. Rai, Roger Feakins, Laura J. Pallett, Derek Manas, Brian R. Davidson

**Affiliations:** 1 Centre of Surgical Innovation, Organ Regeneration and Transplantation, University College London (UCL), London NW3 2QG, UK; b.davidson@ucl.ac.uk; 2 Wellcome/EPSRC Center for Interventional and Surgical Sciences (WEISS), London W1W 7TY, UK; 3 Royal Free NHS Foundation Trust, London NW3 2QG, UK; r.feakins@ucl.ac.uk; 4 Division of Infection and Immunity, Institute of Immunity and Transplantation, University College London, London WC1E 6BT, UK; laura.pallett@ucl.ac.uk; 5 Newcastle Upon Tyne NHS Foundation Trust, Newcastle-Upon-Tyne NE7 7DN, UK; derek.manas@nhs.net

**Keywords:** irreversible electroporation, locally advanced pancreatic cancer, immunotherapy

## Abstract

Locally advanced pancreatic cancer (LAPC) accounts for 30% of patients with pancreatic cancer. Irreversible electroporation (IRE) is a novel cancer treatment that may improve survival and quality of life in LAPC. This narrative review will provide a perspective on the clinical experience of pancreas IRE therapy, explore the evidence for the mode of action, assess treatment complications, and propose strategies for augmenting IRE response. A systematic search was performed using PubMed regarding the clinical use and safety profile of IRE on pancreatic cancer, post-IRE sequential histological changes, associated immune response, and synergistic therapies. Animal data demonstrate that IRE induces both apoptosis and necrosis followed by fibrosis. Major complications may result from IRE; procedure related mortality is up to 2%, with an average morbidity as high as 36%. Nevertheless, prospective and retrospective studies suggest that IRE treatment may increase median overall survival of LAPC to as much as 30 months and provide preliminary data justifying the well-designed trials currently underway, comparing IRE to the standard of care treatment. The mechanism of action of IRE remains unknown, and there is a lack of data on treatment variables and efficiency in humans. There is emerging data suggesting that IRE can be augmented with synergistic therapies such as immunotherapy.

## 1. Introduction

Irreversible electroporation (IRE) is a novel, non-thermal ablative therapy used to treat solid cancers [1]. IRE treatment destroys cancer cells using electricity [2]. It is applied by placing two or more electrodes in and around the cancer. Electrodes can be inserted percutaneously under image guidance, laparoscopically, or through an open approach following a midline laparotomy.

Multiple short pulses of high-voltage electrical current are applied between electrode pairs. The application of an electric field across cell membranes is thought to initiate the formation of nanopores in the lipid bilayer of the tumour cell’s plasma membrane, leading to homeostatic disruption and cell death [3,4]. The mechanism of action of IRE remains controversial. Initial studies reported apoptosis as the main mechanism of cellular death [5], however there is increasing evidence that necrosis also contributes [6]. Apoptotic cell death involves a number of complex intracellular signalling pathways involving mitochondria [7]. The exact mechanism through which IRE induces apoptosis, and whether this is through mitochondrial-mediated pathways, remains to be established.

Unlike other ablative techniques, such as microwave ablation (MWA), cryotherapy and radiofrequency ablation (RFA), IRE is thought to exert its cytotoxic effect without relying on thermal injury [8]. Multiple studies report that IRE spares blood vessels and bile ducts [9,10,11]. Furthermore, IRE is not susceptible to the “heat sink” effect, a phenomenon where blood flowing in vessels adjacent to the cancer being treated prevents the area of ablation from reaching effective temperatures for cellular damage, leaving viable tumour cells [12]; a concern with ablative methods reliant on thermal injury.

Electrical pulses have been known to be cytotoxic since the mid 20th century and have been used in food and water sanitisation programmes [13,14]. In the 1960s Sale and Hamilton reported detecting leakage of intracellular contents following electrical application and postulated that the application of an electrical field resulted in an increase in membrane potential resulting in the loss of cellular integrity [15]. A decade later the term “electroporation” was coined by Neumann and Rosenheck, who demonstrated that the changes in the membrane permeability were temporary and could be reversed [16]. This is now known as reversible electroporation (RE). RE provided a therapeutic route into cells and has been used in recent years to deliver gene therapy (gene electro-transfer) [17] and to target chemotherapeutic agents to cancerous cells (electrochemotherapy) [18].

Irreversible electroporation was largely ignored in oncology until 2005 when Davalos et al. demonstrated that by increasing the electrical field strength and duration of pulses, the changes in membrane permeability were permanent and could therefore induce cell death [19]. In vivo [20,21] and in vitro [22] studies of cancer cells treated with IRE confirmed its cytotoxic effect. Animal studies observing the effect of IRE in healthy organs such as pig pancreas, liver, kidney, and murine liver followed [23,24,25,26,27].

In 2012, Martin et al. published the first human pilot study assessing IRE treatment in pancreatic cancer [28]. Since then, several studies have reported on pancreas IRE [29,30,31,32,33,34,35,36,37,38,39,40,41,42,43]. Unlike liver IRE therapy [44], pancreas IRE therapy has no established protocol. Most studies to date have used 90 pulses per treatment cycle, with each pulse length lasting 70–90 µs and between 1400–2000 V/cm being delivered [45].

IRE therapy for cancer is associated with procedure related complications, the severity of which relate to the site and size of cancer. For pancreatic cancer (PC) the risks are high because the cancer is surrounded by vital structures, such as the coeliac axis and other large blood vessels, and key biliary and pancreatic structures. There is no consensus on how to avoid damage to the adjacent healthy tissues.

IRE treatment protocols used in clinical practice to date have varied [45], and in part have been developed using data from animal studies. However, the animal and human pancreas are significantly different in cellular composition [46] and electrical impedance, which will likely impact IRE efficacy [47]. Similarly, a cancerous pancreas is different in cellular composition and electrical properties compared to a non-cancerous pancreas, which may further impact the success of IRE therapy [48,49]. Variability in current IRE treatment protocols, such as inter-electrode distance, strength of the voltage applied, and the individual electrical properties of the tissue being ablated all have an impact on treatment efficiency and the area of ablation [23,47,50]. Evaluation of the histological changes and clinical benefit following IRE are needed in both human healthy pancreatic and pancreatic cancer tissue, in order to establish appropriate treatment protocols.

A number of retrospective and prospective human clinical studies on pancreas cancer IRE have suggested a survival benefit [29,30,31,33,37,51,52]. However, IRE is not in routine clinical practice in the United Kingdom for a number of reasons. First, evidence from randomised controlled trails (RCT) is awaited, to support the benefits suggested in selected patient cohorts. Second, there is no consensus on the optimal IRE treatment protocol [45] nor the approach needed to protect adjoining pancreatic tissue.

We can compare pancreas IRE therapy with the IDEAL-D framework [53,54], a model developed by expert consensus to describe the stages through which a new medical device should progress (idea, development, exploration, assessment long-term for medical devices). IRE is currently in the nascent stage of 2a [53], as IRE studies have focused on the measurement and comparison of clinical safety, technical, and procedural success through prospective and case series studies. The UK-IRE users group treated 33 patients with pancreatic cancer (85% with pancreatic adenocarcinoma and 12% with neuro-endocrine tumour of the pancreas, median tumour size of 4 cm) between 2014 and 2017 across three UK sites: King’s College Hospital, London; Newcastle upon Tyne Hospital; and Leeds Teaching Hospital. In this unpublished data set patients had a median length of stay in hospital of 5 days and a 30-day survival of 98%, with a single death reported [55]. This preliminary UK data, in addition to data from published cohort studies, justifies the need for a feasibility randomised trial to further investigate pancreas IRE in the UK. Feasibility randomised clinical trials are needed that aim to assess safety, short term clinical outcomes, and patient centred outcomes, such as quality of life, in order to progress to the next stage in the IDEAL-D framework.

The aim of this narrative review is to provide a relevant and comprehensive overview on the clinical outcome of IRE for unresectable locally advanced pancreas cancer, an analysis of mechanistic studies, and the risks and limitations of current therapy.

## 2. Current UK Management of Locally Advanced Pancreatic Cancer (LAPC)

Pancreatic cancer (PC) is a biologically aggressive tumour. It has a five-year survival rate of less than 6% and an incidence that is increasing in the United Kingdom [56]. Pancreatic resection is the only potentially curative treatment. However, only 10 to 20% of patients are eligible for surgery [57]. Even in those selected patients with early-stage disease who are offered surgery, the 5-year survival is only 20% [58].

Approximately 30% of all patients have locally advanced pancreatic cancer (LAPC) at diagnosis and are not considered for surgical resection [59]. In patients with unresectable LAPC there is local involvement of the coeliac axis or its branches, the superior mesenteric artery (SMA), or extensive portal vein involvement precluding resection and reconstruction, but an absence of distant metastases [58]. Currently, the recommended treatment for LAPC is systemic chemotherapy [59,60]. However, despite advances in drug therapy for pancreatic cancer over the last 20 years, overall survival (OS) and progression free survival (PFS) have not significantly improved [61] with a median PFS of just 15 months [62].

### 2.1. Chemotherapy in Unresectable Locally Advanced Pancreatic Cancer (LAPC)

In 2011, Conroy et al. reported the results of the ACCORD11/PRODIGE4 trial, a landmark study on chemotherapy in pancreatic cancer that demonstrated that 5-FluoroUracil, Leucovorin, Irinotecan, and Oxaliplatin (FOLFIRINOX) treatment had a survival advantage over gemcitabine therapy, albeit with a higher toxicity profile (median OS in the FOLFIRINOX group was 11.1 months compared to 6.8 months in the Gemcitabine group, *p* < 0.001) [63]. In the UK, National Institute of Clinical Excellence (NICE) guidelines published in 2018 recommend systemic combination chemotherapy for both locally unresectable and metastatic pancreatic cancer [64]. NICE recommends first-line combination chemotherapy with FOLFIRINOX in otherwise fit patients (Eastern Cooperative Oncology Group (ECOG)) performance status of 0–1) [65]. In patients with a poor baseline, or those unable to tolerate this regime, NICE recommends gemcitabine alone. Despite showing the best objective response in pancreatic cancer, FOLFIRINOX [66,67] is associated with more frequent and severe side effects, including neutropoenia, diarrhoea, and peripheral neuropathy [62]. A systematic review in 2016 reported that LAPC patients treated with FOLFIRINOX had a median PFS of 15 months (range: 3 to 20 months) [62].

Following chemotherapy for LAPC, staging is repeated to assess response and to again review the technical feasibility of surgical resection. In patients with LAPC undergoing first line treatment with FOLFIRINOX a meta-analysis demonstrated that 25.9% underwent resection following chemotherapy and in this cohort, an impressive 78.4% of patients had R0 resection [62]. This is in comparison to the 67% of R0 resection in patients treated with upfront surgery reported by a meta-analysis in 2018 [68]. Studies have reported that neoadjuvant therapy is safe [69,70,71], and two systematic reviews reported that no deaths have been attributed to FOLFIRINOX specifically in the neoadjuvant setting [62,72].

The SCALOP I trial, a randomised multi-centre trial comparing LAPC patients treated with induction chemotherapy (12 weeks of Gemcitabine and Capecitabine) followed either by Gemcitabine and radiotherapy (GEM Rx) or Capecitabine and radiotherapy (CAP Rx). The median OS was 17 and 14 months for CAP Rx and GEM Rx, respectively. Although the study did not report on the exact number of patients that proceeded to surgical resection it demonstrated that 44.5% of patients with pancreatic cancer treated with initial systemic chemotherapy developed distant metastases and 33.3% developed progression of local disease [73]. There is therefore a major unmet need for improved treatment of locally advanced but unresectable pancreas cancer following first line systemic chemotherapy.

### 2.2. IRE Treatment for Locally Advanced Pancreas Cancer

The main objective of IRE in the treatment of LAPC would be to extend survival and prospective and retrospective studies report a median OS of up to 30 months [31]. In addition to improved survival, other possible benefits of treating LAPC with IRE include local control of tumour progression, symptom relief, and improved quality of life (QoL). There are several validated QoL scores that are routinely used in PC [74], but none specifically modified for IRE treatment.

A single-centre prospective study evaluating the QoL pre and post IRE in patients with LAPC demonstrated that IRE had no adverse effects on QoL but failed to show an improvement [75]. It is important to note that the study did not take into account concomitant treatment with chemotherapy after IRE and this may confound the results.

Local treatments may seem an attractive option in the treatment of pancreatic cancer in view of the large number of patients presenting with locally advanced disease. However, it is important to note that PC is often a systemic disease from the earliest stages [76], and therefore local treatments alone are likely to be inadequate. Combination treatments involving IRE in conjunction with chemotherapy and immunotherapy may offer an approach that tackles both the systemic and local impacts of the disease progression.

One consideration for IRE is the timing of treatment in relation to chemotherapy. Studies that have delivered IRE before chemotherapy have shown only a modest increase in median OS [29,36]. Mansson et al. treated patients with IRE as a first line therapy and reported a median OS of 13 months compared to 9.9 month for patients on the National Quality Registry for Pancreatic and Peri-ampullary Cancer registry (*p* = 0.511) [36]. In contrast, studies using IRE after induction chemotherapy offered a greater clinical benefit, with a median OS rate of 27 months, according to a systematic review in 2019 [1].

### 2.3. Immunotherapy in Pancreatic Cancer

Over recent years immune checkpoint blockade agents such as programmed death ligand 1 (PD-L1) and PD-1 inhibitors have been increasingly used as successful therapies for many solid tumours [77]. However, phase I and II clinical trials have so far failed to demonstrate any clinical benefit in patients with pancreatic cancer [78]. The immune resistance observed may be attributed to pancreatic cancer’s tumour microenvironment (TME) [79]. Unlike other solid tumours, where immunotherapy has yielded a successful response, the TME in pancreatic cancer is characterised by desmoplasia, a rigid stroma, with cell components accounting for only 10–30% of the tumour mass [80]. The dense extracellular matrix (ECM) [81] forms a rigid barrier and results in elevated tumour interstitial pressure and reduced vascularization and diffusion [82]. The rigid ECM may act as a physical barrier denying immunotherapeutic agents access to the tumour environment and limiting efficacy. Interestingly, in studies where chemotherapy was combined with immunotherapy the results were more promising, suggesting a possible synergistic effect [83].

## 3. Systematic Review of IRE in Human Pancreas Cancer

A systematic search was performed using PubMed regarding the clinical use and safety profile of IRE on pancreatic cancer in humans for studies published in English from January 2010 to November 2020. Further studies were identified by reviewing appropriate references from review articles. Case reports, studies reporting on less than 10 patients, and those that did not report on median OS were excluded.

## 4. Clinical Outcomes of IRE for LAPC

There have been no prospective randomised trials evaluating the harms and benefits of IRE therapy in LAPC. The survival figures reported for IRE in LAPC are summarised in Table 1. Availability of the new treatment, reflected in the countries that have reported on IRE, demonstrates that it is a technology available across Europe, Asia, and North America. The published median OS range in patients undergoing IRE was from 10–30 months [31,52]. Such variation in OS may be explained by the type of study, the selection criteria used, and expertise and experience of IRE delivery, as well as the variety of neoadjuvant and adjuvant chemotherapy used in combination. The reported data came from studies of patients that were not randomised or controlled. Two studies attempted propensity score matched (PSM) analysis [38,84], a statistical technique that creates a control group [85]. Following PSM, He et al. found that the OS and PFS rates of LAPC patients following neoadjuvant chemotherapy and the use of subsequent IRE treatment were better than that of patients treated with chemotherapy alone (2-year OS rates; 57.9% vs. 18.1%, *p* < 0.001, 2-year PFS rates; 31.4% vs. 7.1%, *p* < 0.001). The promising result for PSM studies suggests that there is potential for IRE to have a beneficial impact in LAPC and warrants the need for further ongoing studies.

One study that reported a significantly lower OS was Mansson et al. [36]; who reported a median OS of only 13 months, similar to the OS reported in the SCALOP 1 trial. In this study there was a delay of 88 days between the date of the radiological diagnosis and IRE treatment, due to waiting times for IRE treatment. The time delay between delivering IRE and chemotherapy, the standard treatment, may be one explanation for the modest OS reported.

It is important to note that there is variability in the maximum cancer size treated. One study reported a survival advantage in patients with tumour sizes less than or equal to 3 cm undergoing IRE; patients with tumour sizes greater than 3 cm had a median OS of 22.7 months, whereas those with a tumour size less than 3 cm had a median OS of 33.8 months (*p* = 0.002) [40]. Other similar clinical studies suggested that IRE is most effective in tumour sizes less than 4 cm [35,37,52,91,92].

The largest dataset published on IRE in pancreatic cancer comes from a Martin et al. [37], who reported on a multiple site experience of pancreas IRE. As one of the first case series reporting pancreas IRE, this was an unstructured review of multiple protocols and provided an initial understanding into the clinical use of pancreas IRE in LAPC. Here, 200 patients across multiple centres were enrolled with radiographic stage III LAPC and excluding borderline resectable disease. IRE was delivered alone (in 150/200) or in combination with pancreatic resection (in the remaining 50/200), where it was used as a tool for margin accentuation; a surgical technique that involves the application of IRE to the cancer in order to increase the chances of an R0 resection. All patients underwent initial treatment with chemotherapy, chemo-radiotherapy, or both, depending on individual institution protocols, and with considerable inter-centre variation in pre-IRE therapy. Patients were subsequently restaged and those free of distant disease were considered for IRE treatment. All IRE procedures were performed via an open approach and involved intra-operative ultrasonography to guide probe placement. The number of electrodes, voltage setting, and spatial organisation of probe placement were determined by the operating surgeon. Importantly, in the patients receiving IRE without resection, 60% were treated with neoadjuvant Gemzar^®^ -based chemotherapy and 29% with a FOLFIRNOX regime. While, 47% of patients received radiotherapy prior to IRE. In the patients that underwent resection followed by IRE for margin accentuation, 43% of patients were treated with Gemzar based chemotherapy, and 38% with FOLFIRINOX prior to resection and IRE delivery. In addition, 52% also received neoadjuvant radiotherapy. The exact radiotherapy protocol was not specified, and 160 patients out of the 200 in the study underwent additional therapy following IRE. This was with a range of adjuvant chemotherapy (67%) regimes or radiotherapy (13%). No information is available regarding the specific radiotherapy treatment protocol.

A median of four probes (range: 2–6 probes), with a median pulse number of 90 (range: 70–200 pulses), were used in the patients receiving IRE without resection. The reported median OS in this group was 23.2 months (range: 4.9–76.1) compared to 28.3 months in patients treated with IRE and resection; not a surprising finding, as resectable disease is likely to represent a smaller tumour burden. Treatment protocols varied between centres and between treatment groups. In patients treated with IRE and resection the median number of probes was two (range: 2–4), delivering a median of 90 pulses (range: 40–200). No attempt was made to evaluate the incidence of positive margins in the resected specimens and how this might compare with resected specimens not treated with IRE. No further analyses were made to determine whether the range of IRE treatment protocols had an impact on the reported results.

## 5. Complications of Pancreas IRE Therapy

IRE is an invasive procedure and is associated with a range of procedure-related complications [1,93]. The average rate of severe complications in the table below (defined as greater than Grade III on the Clavien-Dindo scale [94]) following IRE is 12%, but has been reported to be as high as 42% [87]. The average procedure-related mortality rate is 2% and 0% for open and percutaneous IRE, respectively [95]. A common complication is mild acute pancreatitis [96]. The more serious complications include severe acute pancreatitis, portal vein thrombosis, bile leak, perforations of the gastro-intestinal tract, and pancreatic fistula [30,32,40,52]; summarised in Table 2.

The variability in the reported complication rates across studies may be due to the heterogeneity in treatment protocols used or may be related to the size of the tumours treated. A study by Paiella et al. had one of the lowest complication rates at 10%, with no severe procedure related complications [41]. This study treated patients with tumour sizes less than 4 cm. With their IRE procedure delivering 1500 volts between electrodes placed 1–2 cm apart, with a pulse duration of 70 µs and a total of 90 pulses delivered. In contrast, a study by Narayanan et al. included patients with much larger tumours (up to 8 cm in size) and reported one of the highest rates of complications (a total of 62% of patients developing a procedure-related complication and a severe complication rate of 20%) [40]. The authors did not correlate tumour size with complications. The treatment protocol for this study did not specify the exact voltage applied but reported a higher range using 1500 to 3000 volts, presumably using the highest field strength available commercially (3000 volts) for the largest tumours. Electrodes were placed between 1.8 and 2.2 cm apart and a total of 70 volts each with a duration of 70 µs was delivered.

Other factors contributing to the range of reported complications may be related to the number of years of experience individual centres have with treating patients with IRE, although this information in not always available in the literature. Moreover, the heterogeneity of the rates of reported complications may be related to the method in which IRE is delivered (open vs. percutaneous). The data presented in Table 2 further highlights the need for a robust randomised clinical trial that aims to mitigate for these factors, to better appreciate IRE-associated complications.

## 6. Radiological Response to Pancreas IRE

IRE-treatment response in clinical studies has been determined by radiological imaging, mainly using computerised tomography scanning (CT) and magnetic resonance imaging (MRI). Akinwande et al. prospectively reviewed five LAPC patients who underwent IRE and evaluated contrast enhanced CT images following IRE [97]. They reported that the ablation zone was ill-defined and irregular, without clearly demarcated margins. Immediately following IRE, the ablation zone appeared larger than the original target lesion, thought to be due to the inclusion of both the tumour lesion, as well as the safety margins. Blood vessels within the area of ablation demonstrated narrowing immediately following IRE, which resolved or remained stable in subsequent scans. Subsequent follow-up imaging demonstrated an increased enhancement of the ablation zone, which the authors concluded was due to the formation of granulation tissue and fibrosis, perhaps correlating with the histological findings reported in animal studies. As the surrounding oedema, hyperaemia, and granulation tissue decreased over time, it facilitated the visualisation of the true ablation zone which was smaller than the region seen immediately after IRE, indicating that the ablation zone size immediately following IRE may not be a reliable indicator of the true extent of the area treated, as it is likely to include the surrounding reactive reaction of oedema and hyperaemia. Alternatively, this initial ablation zone may be an important factor in predicting the true ablation zone once the immediate reactive inflammatory process diminishes. There is no consensus on the optimum time post-treatment to measure ablation zone.

**Figure 1:**
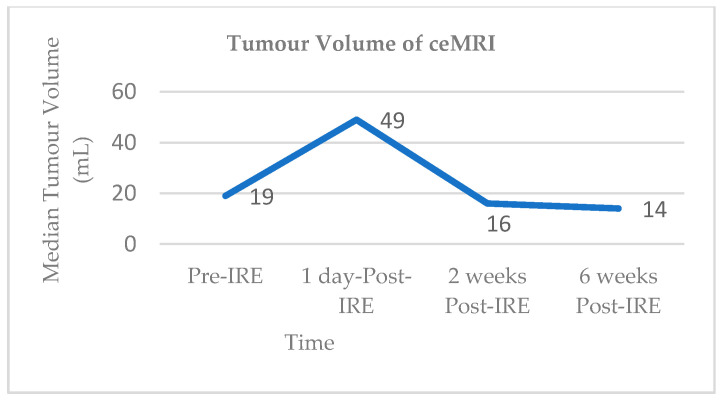
Median tumour volumes on contrast enhanced MRI (ceMRI).

**Figure 2:**
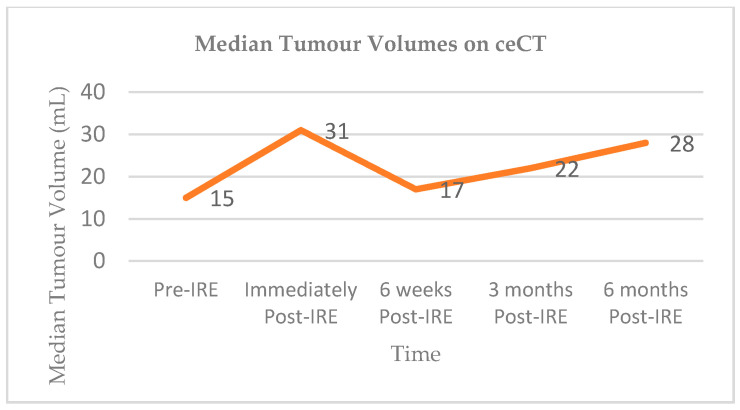
Median tumour volumes on contrast enhanced CT (ceCT).

Vroomen and colleagues assessed imaging characteristics in 25 patients with LAPC following CT-guided percutaneous IRE [98]. All patients had biopsy-proven LAPC and underwent contrast enhanced CT (ceCT) prior to IRE. Subsequent contrast enhanced MRI (ceMRI) was performed 1 day, 2 weeks, and 6 weeks following IRE treatment. At the 6-week mark patients additionally underwent a ceCT. A final ceCT was performed 3 months after IRE. Figure 1 and Figure 2 show the median tumour volumes across the imaging modalities and demonstrate an increase in tumour volume in the initial post-IRE period on both ceCT and ceMRI, followed by a decrease. The authors reported that there was a hyperintense border surrounding the IRE ablation zone in the portal venous phase in 71% of patients, noted 1 day and 2 weeks post IRE, and which was identified in only 29% of patients at the 6 week follow up. The observed hyperintense rim surrounding the ablation zone 1 day post-IRE may represent reactive hyperaemia and oedematous inflammation and still include residual disease. A longer follow-up is required in order to evaluate the exact significance of this radiological characteristic.

## 7. Pathological Response to Pancreas IRE Therapy and Mechanism of Action

### 7.1. IRE Studies Involving Normal Porcine Pancreas

Studies of the histological effects of IRE on healthy pancreas have been performed exclusively on porcine tissues, with the results summarised in Table 3. IRE ablation settings varied extensively between porcine studies, but were similar to the settings used in human clinical practice (90 pulses per treatment cycle, pulse length of 70–90 µs, and a setting range between 1400–3000 V/cm) [45]. In this setting, the use of IRE initially induced active local inflammation, evident from the presence of oedema of the interstitium [23]. IRE of healthy porcine pancreas induces a significant amount of necrosis for up to 7 days after the procedure, independent of treatment setting, followed by the development of fibrosis [24].

### 7.2. IRE in Animal Models of Pancreas Cancer

The histological effect of IRE on pancreatic cancer tissue has been investigated in mouse xenografts bearing human pancreatic cancer cell lines [100,101,102]. Table 4 summarises the studies. Two studies used orthotopic mouse models and implanted human pancreatic cancer cell lines into the pancreas [100,101]. One study used subcutaneously implanted human pancreatic cancer cells [102]. There are conflicting reports regarding apoptosis in pancreatic tumour tissue following IRE [100,101,102]. One study used transmission electron microscopy (TEM) to study the morphological changes in tumour tissue following IRE [102], and euthanised all animals 30 min following the IRE procedure. In the IRE treated tumours there was evidence of both acute coagulative necrosis and chromatin condensation, a hallmark of apoptosis [103]. However, the authors did not evaluate other evidence of apoptosis beyond structural changes and did not report on the structural changes of individual organelles.

### 7.3. Human Models and IRE

There have been no sequential histological analyses of the effects of IRE on human pancreatic tissue because of the risks and ethics of human in vivo tissue sampling. This is an important knowledge gap, as both normal and cancerous tissues differ between animals and humans [48,49]. These differences may impact the response of the tissue to electrical stimuli and ultimately, IRE efficiency. The current treatment algorithms have been developed using data from animal models and may, therefore, have limited clinical applicability. Evidence of the histological changes that occur in human healthy pancreas and pancreatic cancer following IRE are needed in order to establish clinically relevant treatment algorithms. A clear consensus needs to be reached about the impact of IRE on non-neoplastic pancreas adjacent to cancer, neoplastic tissue, and healthy tissue not related to any neoplastic process.

One way to obtain this is information is to apply IRE to perfused human organs (both cancer-containing and healthy). Our previous work on the clinical application of microwave ablation (MWA) to solid tumours demonstrated that a perfused organ could provide different findings, and hence a different treatment algorithm, to that derived from non-viable and non-perfused tissues [104,105,106]. Applying IRE to perfused human pancreas ex vivo, using organs deemed unsuitable for transplant and therefore declined for clinical use may aid the development of an alternative treatment protocol and help define protective criteria for vital structures.

## 8. Pathological Changes in Human Pancreas Cancer Following IRE

**Figure 3:**
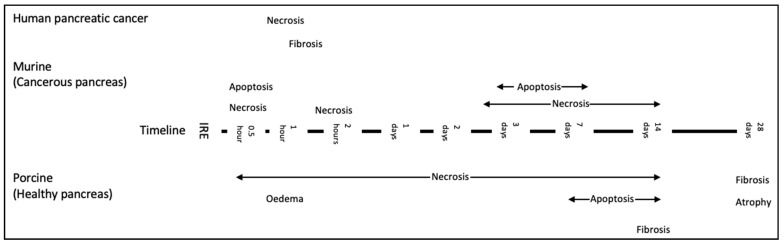
Timeline of the histological changes following pancreas IRE reported in the literature across all models [23,24,100,101,102,109]. Histological data from studies assessing new methods of delivery have not been included in this diagram and are considered separately, please see below.

Information on pathological changes following IRE in human pancreatic cancer tissue is limited to clinical studies that have reported on patients with LAPC or borderline resectable disease that have been downstaged post-IRE, and to patients where IRE has been used as a margin accentuation tool [37,107,108]. Figure 3 demonstrates a timeline of histological changes reported in the literature across different models.

Of the clinical trials discussed above, only five studies reported on the proportion of patients downstaged following IRE and subsequently offered surgical resection [29,30,33,35,40]. This ranged from 5% [33] to 15% [29] across studies with a wide variety of pre-IRE chemotherapeutic regimes. One study also reported on patients who received radiotherapy prior to IRE [40].

Narayanan et al. reported the histological findings on patients who were downstaged in their series [40]. Three of a total of 50 (6%) patients treated with IRE were downstaged to resectable disease and subsequently had surgery. Pathological examination revealed negative resection margins in all three cases and complete tumour necrosis in one [40]. Two resections demonstrated residual viable tumour cells on a background of fibrosis [40]. The patients in this series received chemotherapy prior to IRE treatment and some patients additionally also received radiotherapy before IRE, but the authors did not specify whether the patients who were downstaged were among those who received pre-IRE radiotherapy.

A case report on the use of IRE as an intra-operative margin accentuation tool reported on the histological findings following surgical resection in a patient with pancreatic adenocarcinoma which showed an extensive area of necrosis [109]. Similarly to the animal cancer models, IRE induces necrosis in human cancer tissue. There was no attempt to evaluate the evidence of apoptosis. Therefore, there are no data assessing apoptosis in human pancreatic adenocarcinoma following IRE.

## 9. New Approaches to Pancreas IRE

### 9.1. EUS Guided Pancreas IRE

Lee et al. evaluated the feasibility and safety of applying IRE using endoscopic ultrasound guidance (EUS) in normal porcine pancreas and compared with open IRE [110]. All pigs survived the procedure, and no complications were encountered within 24 h of the intervention. Further to this, the pancreases were resected from all animals for histopathologic evaluation to ascertain the level of apoptosis in the tissue following IRE. Histologically there was evidence of a well-defined area of necrosis affecting the pancreatic parenchyma in both groups. Within the ablation zone, the authors reported apoptotic cell death.

### 9.2. Paddles Rather Than Electrodes for IRE Therapy

Rombouts et al. successfully performed open IRE using paddles rather than needles to deliver the electrical current to healthy pancreas in six pigs [111]. Following euthanasia at 14 days, the porcine pancreas were assessed histologically and demonstrated evidence of fibrosis and lymphocytic infiltration of the ablation zone, new vessel formation, and peripancreatic fat necrosis [111].

## 10. Chemosensitisation Following Electroporation

### 10.1. Reversible Electroporation

Prior to the development of irreversible electroporation as a method of initiating cell death, reversible electroporation (RE) was used to deliver chemotherapeutic drug molecules into tumour cells. The publication of the European Standard Operating Procedures of Electrochemotherapy (ESOPE) in 2006 [112] ushered this treatment modality into standard clinical practise for some cutaneous malignancies [113,114], and advised the use of an electroporator to facilitate the uptake of chemotherapeutic agents. The introduction of bleomycin into skin cancer cells in this manner has been shown to increase cytotoxic potency 1000-fold [18,115] and is currently used in the treatment of cutaneous metastasis from a range of primaries [2]. RE works by electrically inducing transient pores in the lipid membrane [16]. RE treatment protocols have been standardised and involve the application of between 400 to 960 volts across pre-positioned needle electrodes spaced 4 mm apart [116]. No similar standardisation currently exists for pancreas IRE. During IRE treatment, the voltage delivered ranges from 1400 to 2000 volts but can go up to a maximum value of 3000 volts through individual needles that are typically placed between 15 to 20 mm apart [45]. RE has been demonstrated in animal [117], and in vitro pancreatic cancer cell lines models [118], to increase the potency of chemotherapeutic drugs. Preliminary human studies, assessing the impact of delivering chemotherapeutic drugs intraoperatively followed by RE in LAPC patients, have been performed and reported no procedure related mortality and no major post-operative complications [119,120]. However, both studies that showed promising results assessed the impact in a small sample size and neither was randomised.

### 10.2. Irreversible Electroporation

**Figure 4:**
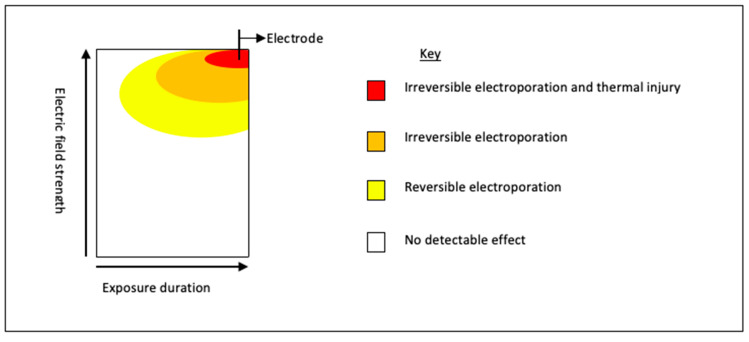
Schematic representation of the basic relationship between electric field strength and length of pulse; key parameters in establishing electroporation. For electroporation to occur, the transmembrane potential difference needs to be between 0.7 and 1 volts [123], once this is achieved, pores form. With increasing voltage and/or increasing length of treatment, these membrane changes become permanent, i.e., IRE resulting in cell death [123].

A number of studies have demonstrated that the area of IRE ablation may be surrounded by a rim of RE [50]. Figure 4 demonstrates the schematic representation of the areas of differing electroporation with increasing voltage and time (adapted from Yarmush et al. [50]). Intra-procedural chemotherapeutic or immunotherapeutic drugs can be taken up by the surrounding area of RE and IRE, and may result in a more robust tumoricidal effect. This effect was reported in a study that observed Gemcitabine levels in the serum, liver, and pancreatic tissue of murine models with orthotopic human pancreatic cancer cell lines [121]. The authors treated mice with either systemic Gemcitabine alone or pancreas IRE delivered between two systemic doses of Gemcitabine. Mice were euthanised and levels of Gemcitabine analysed. The results demonstrated significantly higher concentrations of Gemcitabine in electroporated pancreatic tissue compared to non-electroporated tissue (13,567 ng/mL vs. 4124 ng/mL *p* = 0.0009) [121]; a 3-fold increase in levels. Although an increase was observed in both the serum and hepatic concentration of Gemcitabine in IRE treated animals, suggesting a systemic response this was not statistically significant [121]. The combination of focal tumour necrosis (as a result of ablation and concomitant chemotherapy) with a peripheral sensitivity to chemotherapy would be ideally suited to a cancer such as pancreatic adenocarcinoma, which typically has microscopic seeding to surrounding tissues [122].

An ambitious study, evaluating the effects of IRE and chemotherapeutic agents, gemcitabine, and FOFIRINOX, was recently conducted by Bhutiani et al. [124]. The authors used PDAC human cancer cells lines implanted into the pancreas of nude athymic mice (orthotopic in vivo element) and human pancreatic cancer cell lines (in vitro element). Apoptotic index (AI), survival analysis and histological evaluation was performed. They reported an increase in apoptotic index in pancreatic cancer cells exposed to both chemotherapy and IRE, compared with IRE alone or Folinic acid alone (AI 34.2% IRE  +  FOLFIRINOX vs. 5.2% IRE vs. 4.4% FOLFIRINOX; *p*  =  0.01). This translated into lower tumour mass and significantly improved overall survival of murine models (7 days IRE  +  FOLFIRINOX vs. 4 days IRE vs. 3 days folinic acid; *p*  =  0.026).

In addition to animal models, a phase I safety study has been carried out in five patients with LAPC, who were also treated with IRE and peri-procedural administration of chemotherapy (either FOLFIRINOX or Gemcitabine; the agent used was based on the last regime delivered pre-operatively). IRE was delivered via a mid-line laparotomy. Unfortunately, the authors do not specify the exact IRE settings used. Systemic chemotherapy was delivered intra-operatively, 30 min after the end of the IRE procedures. Short-term safety and clinical outcomes were reported. The median follow up period was 81 days and the authors reported that no patients demonstrated dose-limiting toxicity, disease progression, or procedure related complications or mortality. The authors concluded that IRE and peri-procedural systemic administration of FOLFIRINOX or gemcitabine was well-tolerated and safe at early follow up and suggested that IRE alters the tumour, which augments the chemotoxic effects of the administered drugs.

## 11. Immune Landscape in the Pancreas, in Health and Disease

Many innate and adaptive immune cells contribute to tumour surveillance, but T cells are key mediators of a successful anti-tumour response. In the few patients with pancreatic cancer surviving long-term there were high-quality anti-tumour T cell responses detectable, providing a rationale for harnessing T cells and their functionality for tumour control [125,126]. T cell function is regulated by expression of inhibitory receptors, such as programmed cell death 1 (PD-1) and cytotoxic T lymphocyte-associated antigen 4 (CTLA-4), which when overexpressed limit T cell function. Current immunotherapeutic strategies being used to treat cancer, including “immune checkpoint blockade” strategies, which block such inhibitory receptors, aim to “unleash” a functional anti-tumour immune response. Such therapies have shown promise in many solid tumour types [127]. Nonetheless, their efficacy in patients with pancreatic cancer has been limited, despite showing substantial clinical benefit in murine models [78,128,129].

The ability of T cells to mediate cancer protection depends on the capacity to enter the tumour microenvironment (TME) and persist there. Immunologically, the pancreatic TME is especially suppressive, with a stromal cell network that physically excludes and limits the function of the anti-tumour T cells that are capable of killing cancerous cells. The TME is enriched with other immune cells, which further contribute to the downregulation of successful anti-tumour responses, such as regulatory T cells, tumour-associated macrophages, and myeloid-derived suppressor cells (MDSC) [130]. Many patients with pancreatic cancer also exhibit high levels of PD-L1 and PD-L2, the ligands for the T cell inhibitory receptor, PD-1 [131]. High levels of PD-L1 correlate with reduced T cells and worse prognosis [128,132,133].

A particularly important subset of T cells, termed tissue-resident memory T (T_RM_) cells, reside permanently within tissues, where they are conveniently poised to orchestrate local tumour control. Accumulating evidence is beginning to point towards the integral role T_RM_ play in directly inhibiting tumour growth, making T_RM_ attractive novel therapeutic targets [134]. With these cells in mind, Weisberg et al. [132] described a population of pancreatic CD8^+^ PD-1^+^ T_RM_, capable of the efficient production and secretion of soluble mediators, such as the cytokines IFNg and TNFa with potent anti-tumour activity. Inhibition of the PD-1 pathway significantly enhanced the functional capacity of such T_RM_ [135].

Another attractive immunotherapeutic target that has gained traction recently is the unconventional T cell population, known as gd T cells, due to their potent cytotoxicity and ability to support the function of classical T cells. However, caution must be applied when considering harnessing gd T cells in patients with pancreatic cancer, as although they are highly prevalent in the TME, they express high levels of immunosuppressive checkpoint ligands and limit anti-tumour T cells. In fact, rather than harnessing gd T cells to control tumour cell growth via cytolytic elimination, their deletion or blockade would perhaps be more effective strategy, as evidenced by a recent study in mice. Deletion of pancreatic gd T cells in vivo allowed more CD8^+^ T cells to infiltrate the TME, and thus limited tumour cell growth [136].

## 12. Altering the Systemic Immune Response to Pancreatic Cancer with IRE

As IRE emerges as a novel, non-thermal, image-guided tumour ablation technique for the treatment of pancreatic cancer, it is essential to consider how the process of triggering apoptotic cell death of the tumour impacts both the local and systemic immune response. IRE significantly modifies the structure and composition of the TME, resulting in an altered local inflammatory response and the infiltration of immune cells. Although the number of detailed immunological studies evaluating the direct impact of IRE, or IRE as a combination therapy, in pancreatic cancer is limited, the few studies that have been performed to date are discussed below, along with some future perspectives for novel combination therapies that may improve patient outcome.

To date, the published studies combining IRE with an immunotherapeutic approach have been restricted in scope due to their focus on the systemic immune response and not investigating the local immune response.

One study, by He et al., retrospectively evaluated fluctuations in peripheral immune cell composition by flow cytometry in patients receiving pancreatic IRE for LAPC. In support of a beneficial role for immune cell involvement in the setting of IRE, the authors reported an increase in global CD4^+^ T cells, CD8^+^ T cells, and NK cells in a subgroup of patients surviving longer post therapy [137]. Antigen specificity and cellular migration was not considered however, so whether the increased effector T cells observed are able to enter and persist in the TME, or able to recognise and lyse tumour cells remains unknown. The authors did also note an increase in the systemic levels of some key immunomodulatory cytokines, IL-6 and IL-10 [137], which are known to promote the expansion and differentiation of immunosuppressive myeloid-derived suppressor cells and/or T_REGs_ [138]. However, importantly, no increase in the frequency of circulating T_REGS_ was seen, in fact, a decrease in peripheral T_REG_ frequencies was associated with improved survival [137]. This finding was also reported by Pandit et al., in patients undergoing IRE for pancreatic adenocarcinoma [139]. Therefore, blockade or depletion of T_REGS_ within the TME warrants further investigation, with the aim of improving the functional capacity of tumour-specific T cells [140]. Taken together these findings support the further investigation into studies combining IRE with immune checkpoint blockade, such as anti-CTLA-4 (e.g., Ipilimumab), that can deplete immunosuppressive T_REGs_, or anti-PD-1 (e.g., Pembrolizumab, Nivolumab), which will likely enhance the functionality of both the T_RM_ pool and any infiltrating tumour-specific effector T cells recruited as part of the inflammatory response to the IRE.

Furthermore, in a cancer model study by Zhao et al., the authors hypothesised that IRE would enhance the efficacy of anti-PD-1 therapy by alleviating some of the stromal-cell induced immunosuppression. Intriguingly, treatment with a combination of IRE and checkpoint blockade (anti-PD-1) promoted the infiltration of CD8^+^ T cells, without the additional recruitment of other immunosuppressive cell types (T_REGs_ or MDSC), and prolonged survival. Here, the authors attributed the increased efficacy of anti-PD1 therapy when used in combination with IRE to a reduction in the immunosuppressive components of the stromal cell network. IRE-induced cell death of local pancreatic cells released a number of soluble mediators able to act as “danger associated molecular proteins”, including adenosine triphosphate (ATP) and high mobility group box protein B1 (HMGB1), which can further promote the activation of a functional anti-tumour CD8^+^ T cell response [141].

Taken together, the use of IRE in patients with pancreatic cancer represents a promising approach for enhancing the current clinical efficacy of immune checkpoint blockade.

## 13. Combination Therapies for Pancreatic Cancer: Combining Immunotherapeutic Approaches with IRE

Another immunotherapeutic modality being trialed for the treatment of many cancers is the use of adoptive cell transfer (ACT) therapy, to generate a robust immune-mediated anti-tumour response via the infusion of ex vivo expanded or engineered immune cells. One such approach that has been tested in the context of pancreatic cancer is the adoptive transfer of T cells specific for MUC1, a tumour-associated antigen overexpressed in invasive ductal carcinomas of the pancreas. When adoptively transferred, MUC-1 specific T cells showed strong tumour cytotoxicity, providing a rationale for the use of adjuvant immunotherapy via adoptive cell transfer in the treatment of pancreatic cancer [142,143].

In line with this many studies have shown that adjuvant cellular therapies involving components of the innate immune response, such as NK cells and populations of gd T cells, are promising in the early host defence against some cancers, including pancreatic cancer. In a small study of 40 patients in China, an autologous NK cell infusion was given to patients after undergoing ablative IRE with some success (NCT02718859 https://clinicaltrials.gov/ct2/show/NCT02718859, accessed: 12 February 2021). The combined therapy (IRE and adaptive NK cell transfer) was well tolerated in patients, and particularly in patients with metastatic disease, a synergistic effect was noted, with an overall improvement in the anti-tumour response [144]. Although some improvement to patient QoL and tumour control was noted, its benefit on PFS and OS remains unclear and warrants further investigation.

Similarly, another study, also assessing the efficacy of adoptive NK cell post treatment with pancreatic IRE in patients with LAPC, found adoptive NK cell therapy to be well tolerated, but due to the small sample size, the authors were unable to report any beneficial effect on PRS or OS [145]. As a result, larger prospective randomized controlled trials are needed to corroborate these preliminary studies.

The potential for the existence of a synergistic relationship between IRE and immunotherapy, a relationship that can be harnessed and exploited for the benefit of patients, although exciting, is still poorly understood. Robust pre-clinical and clinical studies will help to elucidate the relationship between these two treatment modalities and if they can be harnessed to improve patient care.

## 14. Discussion and Future Directions

### 14.1. Clinical Outcomes of IRE for LAPC

The improved survival of patients following IRE for LAPC reported in several studies may reflect the increasing experience in administering IRE of clinicians or better IRE technique. Alternatively, another explanation for the reported improvement in OS may be related to the fact that the data so far has not been randomized or controlled, and therefore, as the clinical experience of IRE increased so did the recognition of patients that may be most likely to tolerate the procedure, thus, the increase in OS is a reflection of selection bias. There is a great degree of variability in pre- and post-IRE treatment, and the observed increase in median OS may reflect the improvement in neo-adjuvant and adjuvant treatment regimes. Data from randomised controlled trials is needed. The treatment of unresectable locally advanced pancreatic cancer with percutaneous irreversible electroporation (IRE) following initial systemic chemotherapy: a feasibility randomised control trial (LAP-PIE trial) is due to open in the UK shortly. This multi-centre study aims to measure the feasibility of recruiting patients with LAPC to a trial comparing the addition of pancreas IRE to further chemotherapy. A qualitative study has been incorporated that will evaluate the patient and clinician perspective of the LAP-PIE trial and additionally report on quality of life through validated questionnaires. Another randomised trial currently underway in the United States is the DIRECT study (the direct irreversible electroporation cancer treatment), which aims to report on the impact of IRE treatment on patients with stage III pancreatic cancer [146]. The LAP-PIE and DIRECT studies will provide valuable additional information on the role of IRE in the treatment of LAPC.

### 14.2. Radiological Response to Pancreas IRE

Post-IRE imaging is difficult to interpret and there remains controversy about the accuracy of the traditional imaging modalities used in clinical practice. There is uncertainty surrounding the observed radiological changes and the extent to which these changes correspond to tumour ablation. Moreover, a decrease in viable cell mass, as demonstrated in imaging, may not always reflect a change in tumour size and therefore complete reliance on tumour size does not provide an accurate assessment of tumour response. An alternative measure of post-IRE treatment response is to consider tumour ablation zone sizes in conjunction with other relevant clinical data, such as alterations in enhancement and diffusion and tumour markers. Combination imaging modalities, such as 18 F-Fluorodeoxyglucose (FDG) positron emission tomography (PET) combined with CT or MRI, may provide an alternative assessment of post-pancreas IRE treatment response. PET imaging has been reported to be superior in evaluating the treatment response of neoadjuvant chemo-radiotherapy in pancreatic cancer compared to CT scanning alone [147], however, whether this applies to post-IRE treatment is yet to be elucidated. There are no reliable data currently available to evaluate whether radiological changes can be used as predictive markers and their biological significance remains unknown.

### 14.3. Pathological Changes Following Pancreas IRE Treatment

The optimal IRE treatment protocol for pancreatic cancer tissue is currently unclear [148]. There is also no consensus on how to protect the healthy tissue surrounding the cancer from injury. Data assessing the impact of varying treatment protocols on healthy human tissue would be of great value. Histological evaluation of human cancer tissue and non-neoplastic tissue following IRE is required to establish a treatment protocol that has more robust clinical applicability and to define protective criteria for vital structures. An accurate understanding of the histological findings immediately following IRE could also be used to develop a method of real-time monitoring of treatment efficacy. Perfusion studies using both healthy human pancreas and perfused resected human cancers may help to develop safe and efficient treatment protocols.

In clinical practice, there are several histological grading/scoring schemes that attempt to quantify tumour regression following neoadjuvant therapy for carcinoma of the pancreas. These are based on the presumptive degree of tumour destruction or on the amount of residual tumour. Of course, there is no pre-therapy comparator. Nevertheless, grading the tumour response appears to have prognostic value. These schemes might also be applicable in the setting of IRE, but it is likely that modifications will be required to identify histological responses to IRE that have prognostic relevance.

**Figure 5:**
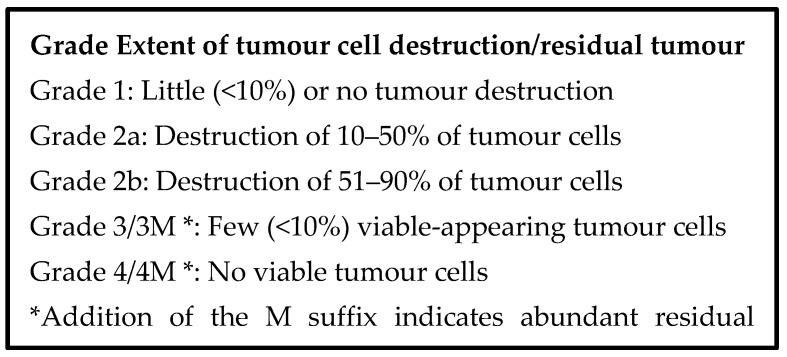
Regression grading scheme proposed by Evans et al. [149].

**Figure 6:**
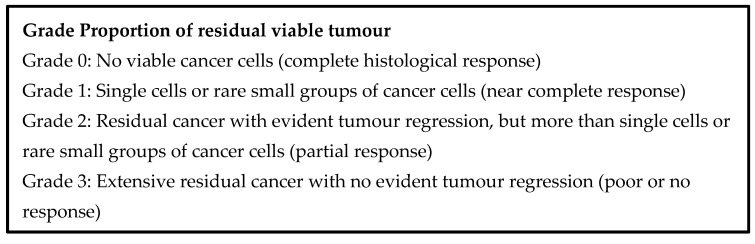
CAP tumour regression grading system.

Two schemes in common use for evaluating neoadjuvant therapy are the Evans scheme and the College of American Pathologists (CAP) scheme [149,150], which are presented in Figure 5 and Figure 6 below. The Evans scheme estimates the presumptive degree of regression from prior treatment based on the percentage area that is occupied by viable tumour in the resection. A suffix “M” denotes the presence of acellular mucin pools, whose significance is currently unknown. The CAP scheme describes, somewhat subjectively, the amount of residual tumour, rather than the degree of regression, and it ignores mucin pools. Many modifications of these schemes and alternative schemes also exist, sometimes attempting, on the basis of clinical evidence, to reduce the number of categories so that the grades become more meaningful, prognostically [151,152,153].

The Royal College of Pathologists’ dataset for pancreatic cancer resections recommends the CAP scheme for clinical practice [153]. An international expert consensus meeting on tumour response scoring also recommended the CAP scheme and favoured assessing residual tumour burden over estimating regression [152].

For the pathologist, extensive sampling is necessary in order to detect any residual tumour. Ideally, this is in the form of large blocks (wholemount blocks) that sample large areas and allow comprehensive assessment. The extent of sampling that is appropriate or necessary to produce reliable information and to allow comparisons between studies is currently unknown [152].

### 14.4. New Approaches to Pancreas IRE

The endoscopic method of IRE application reported by Lee et al. may prove to be a valuable development in the delivery of IRE, particularly in patients where a trans-abdominal approach is precluded, for example in patients with abdominal wall varices. Being a less invasive approach, it may be associated with fewer complications, but does involve deep general anaesthesia and neuromuscular blockade. The risks and benefits will require further evaluation. [154]. Applying IRE through paddles may offer a more homogenous application of an electrical field and be less traumatic than the piercing action of needle-delivery, however, paddle IRE will require an open, more invasive approach and, as previous studies have demonstrated, this approach is associated with more complications compared to minimally invasive approaches [155]. Furthermore, applying IRE via needles may be more advantageous as a range of tumour sizes may be treated by altering the number and configuration of needles, whereas paddle delivery has limited flexibility.

### 14.5. IRE Synergy with Chemotherapy

In vitro studies suggest that IRE may have a chemosensitisation effect in pancreatic cancer cells. Although exciting findings, they are still in the early stages and have yet to be reproduced. The variation in IRE treatment protocols (electrode number, voltage, inter-electrode distance) makes it difficult to assess which treatment parameters will yield the most tumoricidal effect when used in conjunction with chemotherapeutic agents. In addition, it is unknown which chemotherapeutic concomitant treatment will provide the most effective result and whether systemic administration or local administration is more potent. Given the range of variables that can potentially impact the final cytotoxic result, a standardised treatment algorithm would be valuable. This needs to be developed on human tissue (cancer containing and cancer free), with its unique electrical properties [156]. One way to achieve this is to assess the impact of varying IRE treatment variables and cytotoxic drugs using a viable machine perfused ex vivo organ.

### 14.6. Immunological Response to IRE and Synergy with Immunotherapy

IRE may exhibit an immunomodulatory effect by altering systemic T_REGS_ in LAPC [139]. The mechanism through which this immunomodulation occurs is not currently known. One explanation is that IRE induces cell death that leads to the release and recruitment of immune-modulating chemokines. Animal studies suggest that IRE fundamentally alters the TME and causes the release of immune altering molecules, which may act to potentiate existing immunotherapy agents, however this has yet to be reproduced in humans [141]. The potential alteration of the immune microenvironment is yet to be demonstrated, and much about the relationship between IRE and immunotherapy remains unknown. More data are needed to better understand the synergy between the two modalities. Crucially, the animal models to date have used xenografts which are immunodeficient, in order to avoid immune rejection. The absence of a competent immune system may limit the evaluation of immunotherapies in these models. Genetically modified animal models such as the KPC model and its variants [157] may be more appropriate to further our understanding of the synergy between immuno-therapeutics and IRE.

## 15. Conclusions

IRE may herald a new dawn in the treatment of pancreatic cancer. Even more exciting is the potential for the existence of a synergistic relationship between IRE and a concomitant treatment (chemotherapy and immunotherapy). Despite its potential promise, much about IRE remains unknown. New and innovative methods to garner this knowledge include evaluation of IRE in human ex vivo organs using machine perfusion technology and genetically modified animal models. The promise from early uncontrolled studies may just be the dawn of a new field of IRE guided therapies for pancreas cancer.

## Figures and Tables

**Table 1:** Clinical studies evaluating irreversible electroporation (IRE) in pancreatic cancer.

Authors	Date	*n*	Cancer Stage and Size	Median OS (Months)	Location	Type of Study
Veldhuisen et al. [86]	2020	52	LAPC ≤ 4.5 cm	17.2	The Netherlands	Comparative
Ruarus et al. [87]	2020	50	LAPC ≤ 4.6 cm (*n* = 40) Post-surgical local recurrence (*n* = 10)	LAPC: 17 Post-surgery local recurrence: 16	The Netherlands	Prospective single arm
He et al. [84]	2020	32	LAPC > 4 cm included	24	China	Propensity Score Matched analysis
Holland et al. [31]	2019	152	LAPC ≤ 5.5 cm	30	Multi-national	Prospective observational using patient registry
Liu et al. [88]	2019	54	LAPC (*n* = 28) metastatic PC (*n* = 24)	LAPC: 20 metastatic PC: 14	China	Prospective
Flak et al. [30]	2019	33	LAPC ≤ 5 cm	18	Denmark	Prospective single arm
Mansson et al. [36]	2019	24	LAPC ≤ 3.5 cm	13	Sweden	Prospective
Leen et al. [33]	2018	75	LAPC ≤ 5 cm	27	United Kingdom	Prospective
Sugimoto et al. [89]	2018	8	LAPC ≤ 5 cm	24	Japan	Prospective
Huang et al. [90]	2018	70	LAPC≤ 5 cm	22	Taiwan	Retrospective
Vogel et al. [91]	2017	15	LAPC ≤ 5.5 cm	16	The Netherlands	Prospective
Scheffer et al. [42]	2017	25	LAPC ≤ cm	17	The Netherlands	Prospective
Narayanan et al. [40]	2016	50	LAP > 3 cm included	27	United States of America	Retrospective
Mansson et al. [35]	2016	24	LAPC ≤ 4.5 cm	17.9	Sweden	Prospective
Lambert et al. [52]	2016	21	LAPC ≤ 6.5 cm	10	Czech Republic	Retrospective
Martin et al. [37]	2015	200	LAPC	24.9	United States of America	Prospective
Kluger et al. [32]	2015	50	LAPC < 3 cm	12	United States of America	Prospective
Paeilla et al. [41]	2015	10	LAPC	17	Italy	Prospective
Martin et al. [38]	2013	54	LAPC	20	United States of America	Prospective with Propensity Score analysis

Abbreviations: OS; Overall Survival, LAPC; Locally Advanced Pancreatic Cancer, PC; Pancreatic Cancer.

**Table 2:** Incidence and severity of complications reported in clinical studies of IRE treatment for pancreas cancer.

Study	Date	Sample Size	Method of IRE Delivery	All Complications	Severe Complications
Veldhuisen et al. [86]	2020	52	Percutaneous	37%	-
Ruarus et al. [87]	2020	50	Percutaneous	58%	42%
Liu et al. [88]	2019	54	Open (16/54)	19%	1%
Liu et al.	2019	54	Percutaneous (38/54)	44%	3%
Holland et al. [31]	2019	152	Percutaneous	18%	13%
Flak et al. [30]	2019	33	Percutaneous	33%	21%
Mansson et al. [36]	2019	24	Percutaneous	33%	25%
Huang et al. [90]	2018	70	Open	23%	4%
Leen et al. [33]	2018	75	Percutaneous	25%	8%
Zhang et al. [92]	2017	21	Percutaneous	19%	0%
Sheffer et al. [42]	2017	25	Percutaneous	48%	-
Vogel et al. [91]	2017	15	Percutaneous	53%	-
Narayanan et al. [40]	2016	50	Percutaneous	62%	20%
Mansson et al. [35]	2016	24	Percutaneous	46%	13%
Lambert et al. [52]	2016	21	Percutaneous	24%	-
Yan et al. [43]	2016	25	Open	36%	8%
Paiella et al. [41]	2015	10	Open	10%	0%
Belfiore et al. [29]	2015	20	Percutaneous	10%	0%
Martin et al. [37]	2015	200	Open + resection (50/200)	40%	-
Martin et al.	2015	200	In situ (150/200)	36%	-
Kluger et al. [32]	2015	50	Open	46%	20%
Martin et al. [38]	2013	54	Open	39%	-
Martin et al. [28]	2012	27	Open	25%	7%

**Table 3:** Histological changes at specific IRE setting and time course following IRE in normal porcine pancreas [23,24,99].

Time	Electrode Type	Probe No	Inter-Electrode Distance (mm)	Pulse No	Pulse Length (ms)	Voltage (Volts)	Ablation Zone (mm^2^)	Histology
1 h	Monopolar	2	10	90	90	1900	Not stated	Oedema Necrosis
2 h	Monopolar	2	10	90	100	1500	240	Necrosis
24 h	Monopolar	2	10	70	70	2000	134	Necrosis
24 h	Bipolar	1	7	90	70	2100	271	Necrosis
48 h	Monopolar	2	9	90	100	1500	252	Necrosis
72 h	Monopolar	2	20	90	100	3000	Not stated	Necrosis
7 days	Monopolar	2	10	90	90	1900	Not stated	Apoptosis Necrosis
7 days	Monopolar	2	15	90	70	2700	Not stated	Necrosis
14 days	Monopolar	2	10	90	100	1500	<100	Fibrosis
14 days	Monopolar	2	15	90	100	1500	Not seen	No changes
14 days	Monopolar	2	15	70	100	2250	Not seen	No changes
14 days	Monopolar	2	10	70	70	2000	Not seen	No changes
14 days	Not specified	-	Not specified	100	100	2500	Not stated	Necrosis
14 days	Monopolar	2	15	90	100	2300	Not stated	Necrosis Apoptosis
28 days	Monopolar	2	15	90	100	2500	21	Atrophy Fibrosis
28 days	Bipolar	1	7	90	70	2400	207	Atrophy Fibrosis

**Table 4:** Histological changes at specific IRE settings and time course following IRE to murine pancreatic cancer models [100,101,102].

Time	Electrode Type	Probe No	Inter-Electrode Distance (mm)	Pulse No	Pulse Length (ms)	Voltage (Volts/cm)	Ablation Zone (mm^2^)	Histology
0.5 h	Not specified	-	Not specified	64	100	1250	Not stated	Necrosis Apoptosis
24 h	Not specified	-	Not specified	100	100	2500	Not stated	Necrosis No evidence of apoptosis
24 h	Not specified	-	5	90	100	800	Not stated	Necrosis Apoptosis
72 h	Not specified	-	5	90	100	800	Not stated	Necrosis Apoptosis
7 days	Not specified	-	Not specified	100	100	2500	Not stated	Necrosis No evidence of apoptosis
7 days	Not specified	-	5	90	100	800	Not stated	Necrosis Apoptosis
14 days	Not specified	-	Not specified	100	100	2500	Not stated	Necrosis No evidence of apoptosis

## Data Availability

No new data were created or analysed in this study. Data sharing is not applicable to this article.
